# Dosage and Administration-Related Emotional and Clinical Outcomes of Buprenorphine Treatment for Opioid Use Disorder: A Systematic Review and Meta-Analysis

**DOI:** 10.3390/healthcare14182995

**Published:** 2026-09-14

**Authors:** Yunyi Xiao, Miguel Ribeiro Ramos, Paul Montgomery-Marks

**Affiliations:** 1 School of Politics and Law, Hanshan Normal University, Chaozhou 521041, China; 2 School of Social Policy, University of Birmingham, Birmingham B15 2TT, UK

**Keywords:** administration route, buprenorphine, dose–response, illicit opioid use, long-acting formulations, meta-analysis, negative emotional outcomes, opioid use disorder

## Abstract

**Background:** The evidence is still sparse on the effects of various doses and routes of buprenorphine on emotional and clinical outcomes for individuals with OUD. **Methods:** A systematic review and meta-analysis of RCTs was performed following PRISMA and Cochrane guidelines. The electronic databases and other sources were searched for studies that were published from February 2013 to September 2025. Negative emotional symptomology, illicit opioid use, treatment retention, and adverse events were eligible outcomes. Standardized mean differences and odds ratios were used to summarize continuous and binary outcomes, respectively. **Results:** There were five RCTs that met the inclusion criteria. In studies comparing single-dose buprenorphine, a single dose of 96 mg was more effective than a single dose of 32 mg on negative emotional symptom scores (SMD = −0.66, 90% CI [−1.00, −0.32]). But limited evidence suggested that the odds of observing illicit opioid use were lower with subcutaneous buprenorphine than sublingual buprenorphine (OR = 0.61, 90% CI [0.39, 0.94]). There were no apparent differences between dose comparisons or administration-route comparisons with respect to adverse events. Levels of certainty of evidence were relatively low. **Conclusions:** Some limited data suggest there may be a correlation between the dose and route of buprenorphine administration and short-term emotional and illicit opioid-use outcomes. But the number of trials, the size of the samples, and methodological flaws significantly limit the validity and applicability of these results. It is worth emphasising that the high-dose comparisons featured in this review were from single doses in acute treatment settings, and thus are not suitable for extrapolating to everyday, maintenance doses.

## 1. Introduction

Opioid use disorder (OUD) is a significant worldwide health issue that poses a significant risk to morbidity, mortality, and broader social damage [1]. The ever-increasing trend of opioid misuse and overdose-related deaths in most countries has strengthened the necessity of treatment approaches that are not only effective in lowering illicit opioid use, but also able to contribute to the overall clinical and psychological stabilisation in the recovery process.

Pharmacological treatment has been one of the main aspects of OUD care. Buprenorphine is one of the most common medications that is used due to its effectiveness in reducing withdrawal symptoms, craving suppression, and aiding in treatment attendance. Its partial agonist nature and ceiling effect could also have a safety benefit in certain treatment settings [2,3,4]. Nevertheless, despite the proven efficacy of buprenorphine in OUD management, there are still crucial gaps in the understanding of how dosage and route of administration variations can affect treatment outcomes. 

The previous literature and reviews have largely concentrated on the traditional clinical outcomes, which include treatment retention, withdrawal control, and illicit opioid use. Although these indicators are essential, they do not fully reflect the overall treatment experience of individuals with opioid use disorder (OUD), who often experience significant psychological distress, emotional instability, and symptoms of anxiety or depression during treatment and recovery. In this regard, emotion-related outcome measures are clinically significant, as difficulties with emotional regulation may influence treatment adherence, symptom burden, and overall emotional stability.

Recent studies indicate that differences in buprenorphine dose are not only related to the results of addiction but also to negative emotional states and other psychological manifestations [5,6,7]. Similarly, the administration route can impact the treatment experience through adherence, convenience, privacy, and continuity of therapeutic effect, especially in scenarios where long-acting subcutaneous preparations lead to a reduction in the reliance on daily dosing schedules [3,8]. However, conclusions made in various studies are conflicting, and heterogeneity in study designs, dosage comparisons, and outcome measurements has complicated making clear conclusions on the extent to which dose-related and administration-related differences are related to mood-related as well as clinical outcomes [7,9].

Past systematic reviews, especially Mattick et al. [7], have mainly assessed buprenorphine maintenance treatment against placebo or methadone and examined traditional outcome measures like treatment retention and illicit opioid use suppression. The current review builds on this body of literature and specifically addresses differences in both emotional and clinical outcomes related to dose and administration in more recent randomised evidence.

Although methadone is an important and well-established intervention for the treatment of opioid use disorder (OUD), the evidence meeting the inclusion criteria for this review primarily consists of comparative studies of buprenorphine interventions. Based on this, this review specifically highlights dose-related and administration-related effects of buprenorphine, although it has an overall interest in pharmacological treatments of OUD.

The purpose of this systematic review and meta-analysis was to review evidence to compare dose-related and administration-related emotional and clinical outcomes for buprenorphine in the treatment of OUD. Negative emotional symptoms, illicit opioid use, treatment retention, and adverse events were of particular interest. Also explored in the review was the question of whether there are any consistent patterns of dose-related and administration-related findings in the available evidence. Some of the trials included high single doses of buprenorphine in acute treatment settings. These comparisons were therefore considered experimental therapies for the short term, and not as evidence for routine long-term high-dose maintenance treatment.

## 2. Methods

### 2.1. Registration

This review was prospectively registered with the International Prospective Register of Systematic Reviews (PROSPERO; registration number: CRD420261353083). It was conducted and reported in accordance with the PRISMA 2020 statement [10] (Supplementary Materials) and relevant guidance from the Cochrane Handbook for Systematic Reviews of Interventions [11].

### 2.2. Eligibility Criteria

Studies that met the following criteria were eligible for inclusion: randomized controlled trials featuring participants with a diagnosis of opioid use disorder and/or opioid dependence that directly compared different doses or routes of administration of buprenorphine and provided extractable data for at least one of the prespecified emotional or clinical outcomes. However, the evidence ultimately met the inclusion criteria primarily related to comparisons involving buprenorphine. Characteristic outcome measures meeting the inclusion criteria should include treatment adherence, illicit opioid use, mood or psychological indicators, health-related outcomes, and urine toxicology test results. Only articles published in English were included.

Studies were excluded if they were animal studies, did not provide pertinent or extractable outcome data, or had a non-randomised or retrospective design. They also excluded studies that were duplicates, interim reports, abstracts of a conference without the full text, and those that were only loosely related to the review question. If the study design or analysis is unable to effectively address such situations, trials involving participants with serious comorbidities, which may significantly alter treatment outcomes, were excluded.

### 2.3. Search Strategy

The following electronic databases were searched: the Cochrane Central Register of Controlled Trials (CENTRAL), Embase, PubMed, Web of Science, and APA PsycNet. Other sources were also searched, such as Current Contents, APA PsycNet, CORK, the National Institute on Drug Abuse (NIDA), the National Drug and Alcohol Research Centre (NDARC), grey literature databases, Dissertations and Theses, Dissertation Abstracts International, COPAC, PolicyFile, and SIGLE. We searched grey literature resources to reduce the risk of publication bias and to identify potentially qualified studies that may not have been published in traditional peer-reviewed journals. Clinical trial registries were also searched to identify ongoing, unpublished, or otherwise difficult-to-locate trials. No additional eligible studies were identified through these registry searches. A systematic literature search was conducted through February 2013 because this review was intended as an extension of Mattick et al. [7], who had conducted a systematic search through January 2013. The search therefore started in February 2013 to avoid duplicating the previous search period and to maintain continuity with the previous search. The keywords were combinations of keywords and subject headings pertaining to opioid use disorder, buprenorphine, methadone, placebo, and randomised controlled trials. The full search strategies for each database are provided in Appendix A.

### 2.4. Outcomes

Primary outcomes were treatment retention, illicit opioid use, and emotional or psychological outcomes, including anxiety, depressive symptoms, suicidal ideation, and negative emotional symptoms, if applicable. Because the tools and scales used to assess relevant emotional outcomes differ, the raw mean differences between trials may not be comparable. To synthesise the analysis of continuous emotional outcomes, we used standardised mean differences (SMD), which measure effects based on universal standardised scales.

To assess the use of illicit opioids, the authors referenced evidence from objective methods such as urine analysis and considered subjective self-reports if applicable [12]. Additional outcomes included adverse events, mortality, and other health-related outcomes. Outcomes were relevant to treatment effectiveness and were based on measures used in other opioid substitution therapy studies and reviews [7].

### 2.5. Study Selection and Data Extraction

Titles and abstracts were initially screened by Y.X. against the predefined eligibility criteria. Full-text articles were then assessed, and reasons for exclusion were documented. Data on study design, participant characteristics, interventions, doses, routes of administration, outcomes, and follow-up duration were extracted by Y.X. Hongyi Lin assisted with the organisation of the study records and the verification of selected screening decisions and extracted data. Any uncertainties found during the selection of studies or extraction of data were cleared by discussion. If needed, the Methodological review was provided by M. R. R. and P. M. M.

### 2.6. Risk of Bias and Certainty Assessment

Risk of bias was assessed using the Cochrane Risk of Bias 2 (RoB 2) tool (version 22 August 2019) for randomisation, deviations from the intended intervention, missing outcome data, measurement of the outcome, and selection of reported results [11]. Based on the GRADE system, the strength of evidence for the primary outcomes was evaluated in terms of risk of bias, inconsistency, indirectness, imprecision, and publication bias. Summary-of-Findings tables were prepared for the main results. Risk-of-bias judgements were reviewed under the supervision of M.R.R. and P.M.-M. and any uncertainties were resolved through discussion.

### 2.7. Statistical Analysis

Meta-analyses were carried out for binary and continuous outcomes if sufficient quantitative data were available. Odds ratios were summarised for binary outcomes; for continuous outcomes measured using different scales, standardised mean differences were used. All pooled effect estimates were reported with a 90% confidence interval, in line with the exploratory nature of this review that was prespecified. According to the Cochrane Handbook, confidence intervals can be obtained with varying degrees of confidence, such as 90%, 95%, and 99% confidence [13]. Thus, importance was placed on the size and accuracy of the effect estimates, rather than on the dichotomous thresholds of statistical significance. The 90% confidence interval was retained because it was specified a priori as part of the exploratory analytical framework of the review. This level of confidence was used throughout the analyses and was not based on individual findings of statistical significance. The use of the 90% confidence intervals was not intended to be definitive evidence of treatment effectiveness, but rather to assist in the initial estimation of potential effect patterns in the small and limited evidence base. The I^2^ statistic was used to evaluate statistical heterogeneity. Because few studies contributed to each analysis, heterogeneity estimates were interpreted with caution. Random-effects or fixed-effect models were used based on clinical and methodological characteristics of the included studies and the assumptions underlying each comparison. The results were considered exploratory and hypothesis-generating, with a focus on the width of the confidence intervals, the number of trials included, and the certainty of evidence. In cases where numerical summary statistics for use in the meta-analysis were not explicitly reported, they were calculated or converted from other information contained in published reports by applying methods outlined in the Cochrane Handbook [11]. This approach concerned missing or incompletely reported summary statistics rather than participant-level missing outcome data. Where participant-level outcome data were missing because of attrition or incomplete follow-up, these data were not imputed by the review authors and were instead considered in the risk-of-bias assessment. No additional unpublished participant-level data were obtained from study authors.

## 3. Results

### 3.1. Study Selection

**Figure 1:**
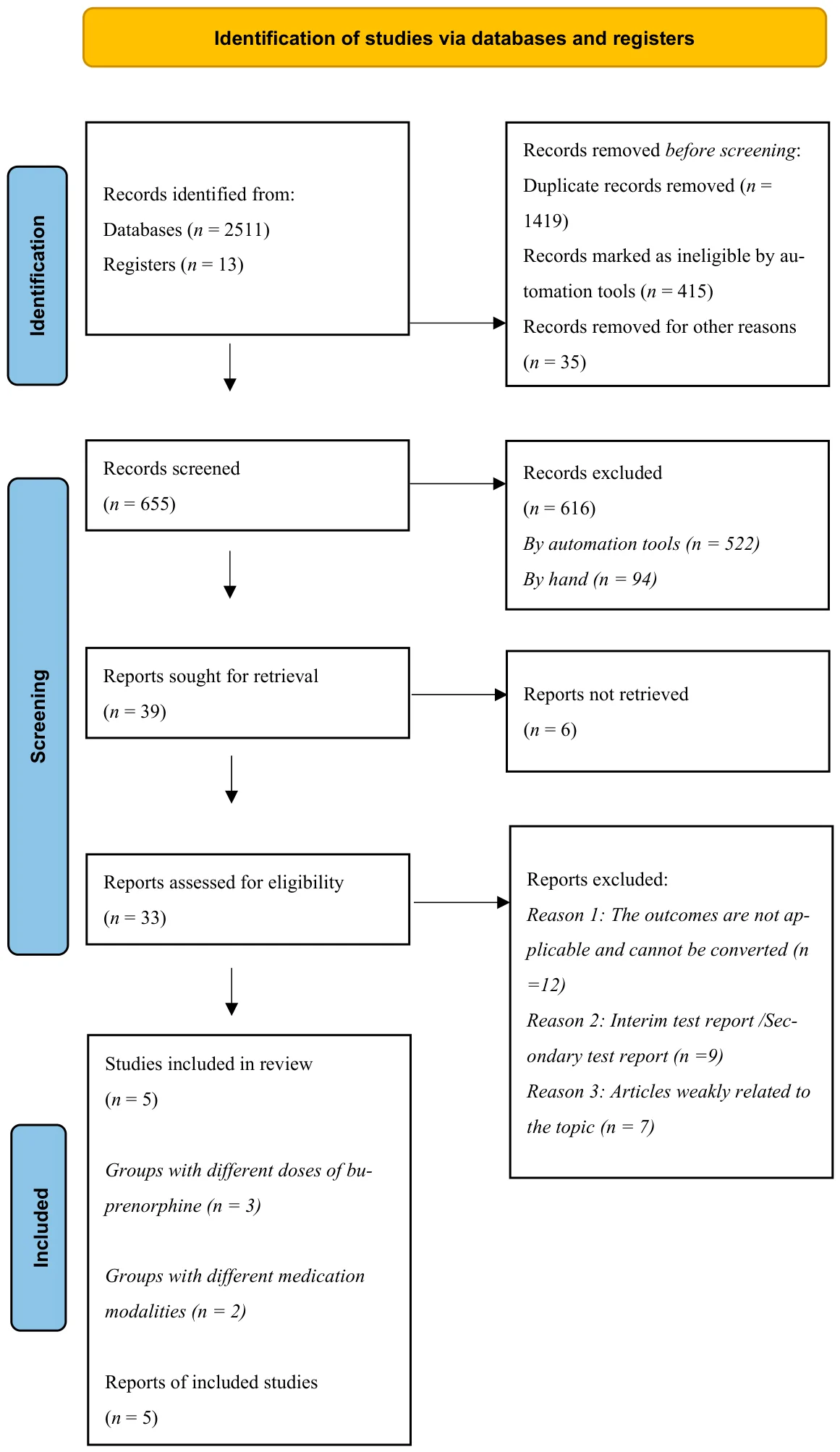
PRISMA flow diagram of study identification, screening, eligibility assessment, and inclusion.

The process of study selection followed the PRISMA guidelines and is shown in Figure 1. In total, 2524 records were first explored by accessing databases. Having filtered the randomised controlled trials and removing animal studies, 522 records were eliminated.

Screening of the titles and abstracts followed, and records that were not related to opioid pharmacological treatment were eliminated. After the removal of duplicates (*n* = 6), 33 studies remained to be evaluated in full text.

After full-text screening, studies were filtered out for a variety of reasons: the absence of similar outcome measures (*n* = 12), inadequate or incomprehensible experimental design (*n* = 9), and irrelevance to the research question (*n* = 7).

Five studies were selected based on the inclusion criteria and were included in the final analysis. Among them, three studies analysed the effects of various dosages of buprenorphine, and two studies analysed various ways of administration.

### 3.2. Characteristics of Included Studies

Five randomized controlled trials met the eligibility criteria. Three studies evaluated different single doses of buprenorphine, but the other two studies compared different routes or formulations of buprenorphine administration.

The trials included in this review were widely varied in the emotional and clinical outcomes measured, the instruments used to measure the outcomes, and the time points at which outcomes were assessed. These are summarised in Table 1.

The emotional and psychological outcomes were not assessed with a common instrument, as mentioned in Table 1. Craving for opioids was measured by the VAS, suicidal ideation by the BSSI, and anxiety by the HAM-A in the dose-comparison trials, while the administration-route trials employed more general clinical- and patient-reported outcome measures. This variation in outcome constructs and measurement methods was taken into consideration in the interpretation of the pooled estimates.

### 3.3. Risk of Bias in Included Studies

The Cochrane RoB 2 instrument covers the five bias domains of the Randomization process, Deviations from intended interventions, Missing outcome data, Measurement of the outcome, and Selection of the reported result.

In summary, the included trials were mostly assessed to be at low risk or to have some concerns for the randomisation process, with some trials not reporting allocation procedures completely. Lintzeris et al. [8] was an open-label study, which led to concerns about the fidelity of the intervention and the reporting of patient-reported outcomes. In some of the studies, there was also a concern about missing outcome data due to attrition or incompletely reported data. It was difficult to determine the risk of bias in the selection of the reported results when protocols or prespecified analysis plans were not available.

**Figure 2:**
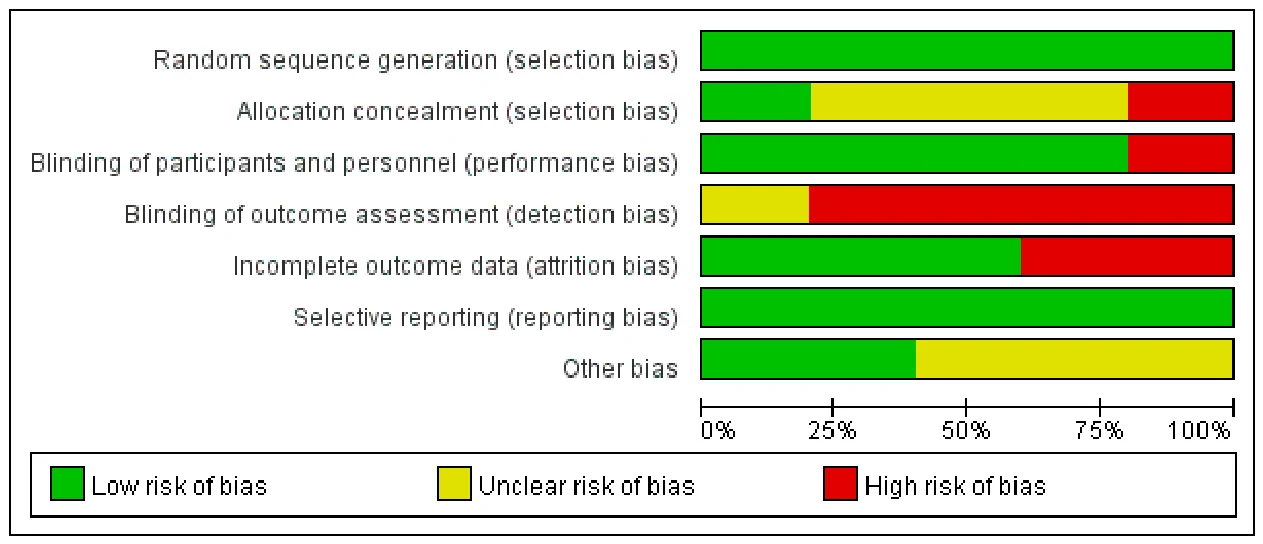
Risk of bias assessment for included studies.

**Figure 3:**
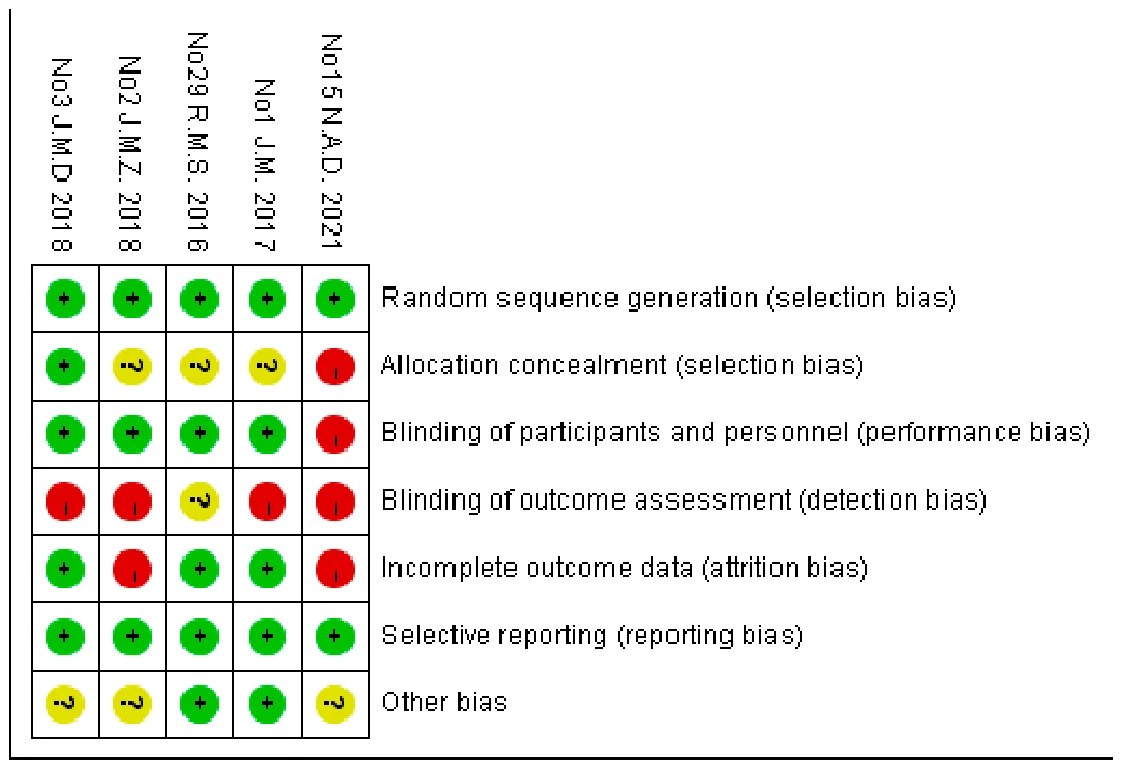
Risk of bias summary of the five included studies [5,6,8,14,15]. Symbols and colours indicate the risk-of-bias judgements: green (+), low risk; yellow (?), some concerns; and red (−), high risk.

Graphs of overall risk-of-bias judgements are shown in Figure 2 and Figure 3. When interpreting the findings and when grading the certainty of evidence, these concerns were considered.

The implications of these sources of bias are important to consider when interpreting the findings. Reporting subjective patient-reported outcomes such as emotional symptoms might be affected by the treatment experience, adherence, and knowledge of treatment allocation, especially in open-label trials. Also, if allocation concealment is not reported as complete, there is some uncertainty about whether allocation to groups was sufficiently protected from selection bias. If there is missingness of outcome data, this may also impact effect estimates if the missingness was different between treatment groups, as could be seen with attrition. These considerations were made when interpreting pooled estimates and led to a downgrade of certainty of evidence.

### 3.4. Single 96 mg Versus Single 32 mg Dose

#### 3.4.1. Baseline Comparability

**Figure 4:**
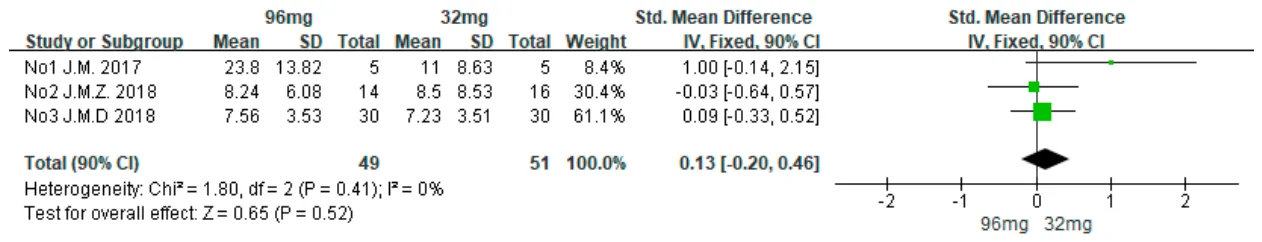
Forest plot of high- vs. low-dose buprenorphine on treatment outcomes [5,6,14]. Squares represent the effect estimates for individual studies, with square size reflecting study weight; the diamond represents the pooled effect estimate, with its width indicating the 90% confidence interval.

The negative emotional indicators were used to compare baseline characteristics between the 96 mg and 32 mg groups of buprenorphine, as shown in Figure 4.

The pooled baseline estimate was close to the null, and the confidence interval included no difference (SMD = 0.13, 90% CI [−0.20, 0.46]), suggesting broadly comparable baseline measurements between the groups.

The heterogeneity was low (I^2^ = 0%), which means that baseline measurements are consistent.

#### 3.4.2. Negative Emotional Outcomes: 96 mg vs. 32 mg

**Figure 5:**
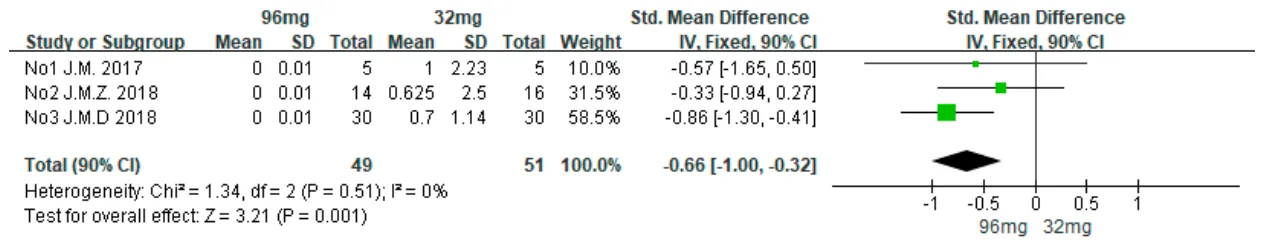
Forest plot of high- vs. low-dose buprenorphine on negative mood indicators [5,6,14]. Squares represent the effect estimates for individual studies, with square size reflecting study weight; the diamond represents the pooled effect estimate, with its width indicating the 90% confidence interval.

As shown in Figure 5, the pooled analysis favoured a single 96 mg dose over a single 32 mg dose for negative emotional outcomes (SMD = −0.66, 90% CI [−1.00, −0.32], *p* = 0.001). Participants assigned to a single 96 mg dose showed a greater reduction in measured negative emotional symptom scores than those assigned to a single 32 mg dose. Statistical heterogeneity was low (I^2^ = 0%). However, because the analysis was based on a small number of trials, the absence of observed heterogeneity should be interpreted cautiously.

Overall, the pooled estimate favoured the single 96 mg dose for the measured negative emotional outcomes. This comparison was for a single administration in an acute treatment setting. However, it does not provide evidence to support high-dose maintenance prescribing.

#### 3.4.3. Adverse Events: 96 mg vs. 32 mg

**Figure 6:**
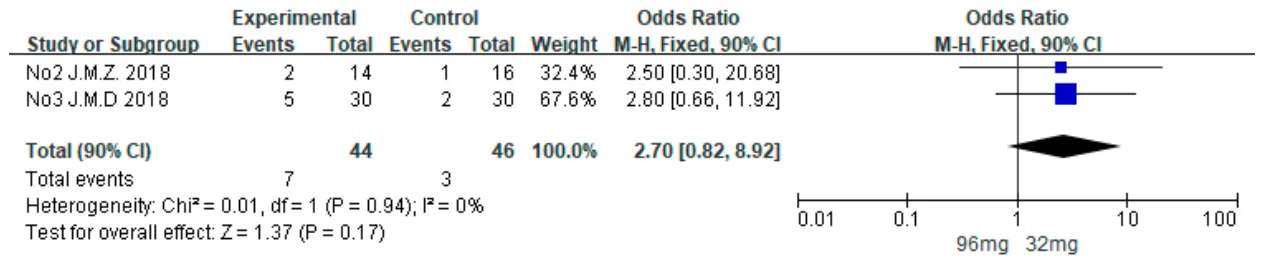
Forest plot of odds ratios comparing single 96 mg vs. 32 mg buprenorphine for adverse events [6,14].

**Figure 7:**
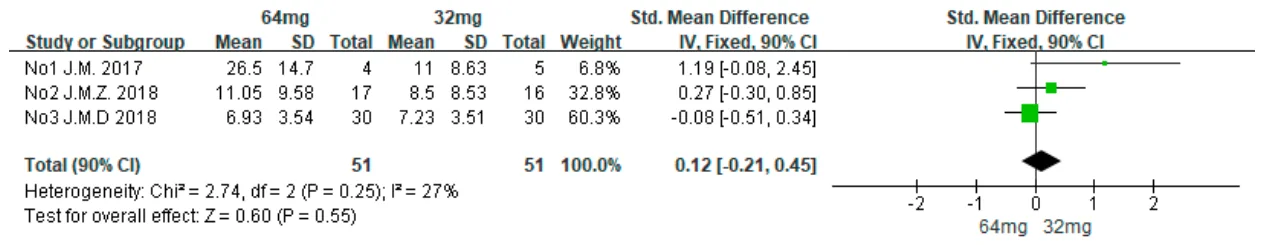
Forest plot of standardised mean differences comparing single 64 mg vs. 32 mg buprenorphine at baseline [5,6,14]. Squares represent the effect estimates for individual studies, with square size reflecting study weight; the diamond represents the pooled effect estimate, with its width indicating the 90% confidence interval.

As shown in Figure 6 and Figure 7, adverse events were reported in the single 96 mg group and 3 in the single 32 mg group. The pooled analysis produced an odds ratio of 2.70 (90% CI [0.82, 8.92]). The wide confidence interval included the possibility of little or no difference, as well as a potentially increased risk of adverse events in the single 96 mg group. We did not find any statistical heterogeneity (I^2^ = 0%). However, there was not much reporting of adverse events, and the estimate was very imprecise. Evidence was therefore insufficient to determine whether adverse-event risk was different between the single 96 mg and single 32 mg dose groups, and safety could not be compared.

### 3.5. Single 64 mg Versus Single 32 mg Dose

#### 3.5.1. Baseline Comparability

As shown in Figure 6, the pooled analysis indicated no clear baseline difference between the single 64 mg and 32 mg groups (SMD = 0.12, 90% CI [−0.21, 0.45], *p* = 0.55), suggesting that the groups were broadly comparable before treatment. Heterogeneity was low (I^2^ = 27%, *p* = 0.25), indicating that the study results showed only mild variability. Therefore, it is considered appropriate to use a fixed-effects model.

#### 3.5.2. Negative Emotional Outcomes: 64 mg vs. 32 mg

**Figure 8:**
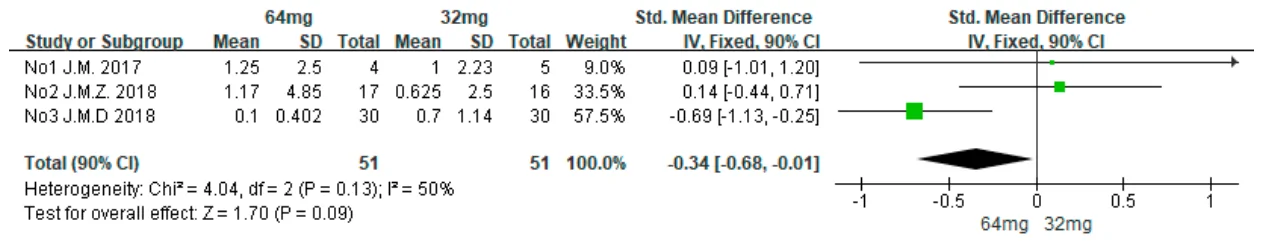
Forest plot of standardised mean differences comparing single 64 mg vs. 32 mg buprenorphine on negative mood indicators [5,6,14]. Squares represent the effect estimates for individual studies, with square size reflecting study weight; the diamond represents the pooled effect estimate, with its width indicating the 90% confidence interval.

As shown in Figure 8, the pooled analysis favoured a single 64 mg dose over a single 32 mg dose for negative emotional outcomes (SMD = −0.34, 90% CI [−0.68, −0.01], *p* = 0.09). However, only a small number of trials contributed to the analysis, and the estimated difference was modest. There was some statistical heterogeneity (I^2^ = 50%). A fixed-effect meta-analysis was used because the studies covered in the analysis assessed the same dose comparison in largely similar acute treatment settings. The heterogeneity estimate was interpreted with caution as a few studies contributed to it. There was an overall preference for the single 64 mg dose, but the overall evidence was uncertain and should be interpreted with caution.

#### 3.5.3. Adverse Events: 64 mg vs. 32 mg

**Figure 9:**
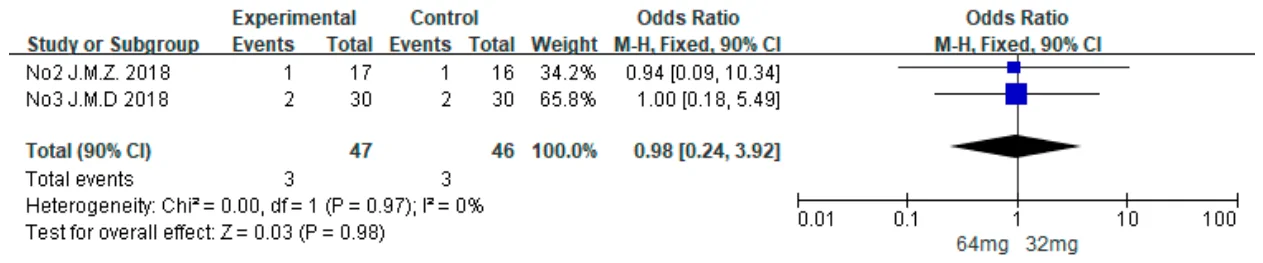
Forest plot of odds ratios comparing single 64 mg vs. 32 mg buprenorphine for adverse events [6,14]. Squares represent the effect estimates for individual studies, with square size reflecting study weight; the diamond represents the pooled effect estimate, with its width indicating the 90% confidence interval.

In each dose group, 3 adverse events were reported, as seen in Figure 9. The pooled analysis produced an odds ratio of 0.98 (90% CI [0.24, 3.92], *p* = 0.98). This confidence interval was wide and could have included the possibility of a lower risk of adverse events as well as a higher risk. However, there was low statistical heterogeneity (I^2^ = 0%, *p* = 0.97). However, the estimate was imprecise, with only a few events being reported. Due to insufficient information, it was not possible to determine the difference in risk of adverse events between the single 64 mg and single 32 mg dose groups.

### 3.6. Administration-Route Comparisons

#### 3.6.1. Illicit Opioid Use

**Figure 10:**
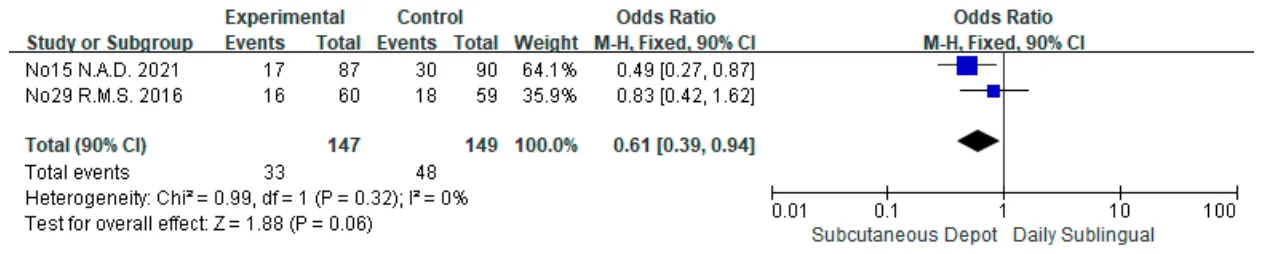
Forest plot of odds ratios comparing long-acting buprenorphine formulations with sublingual buprenorphine for illicit opioid use [8,15]. Squares represent the effect estimates for individual studies, with square size reflecting study weight; the diamond represents the pooled effect estimate, with its width indicating the 90% confidence interval.

As shown in Figure 10, 22.45% of participants receiving long-acting buprenorphine formulations continued to use illicit opioids, compared with 32.21% of those receiving sublingual buprenorphine. The pooled analysis produced an odds ratio of 0.61 (90% CI [0.39, 0.94], *p* = 0.06). The point estimate suggested that long-acting buprenorphine formulations (e.g., those under the skin or in an implant) were preferable to sublingual buprenorphine. However, there were some differences in the interventions’ formulation and delivery, and the pooled estimate should be interpreted with caution.

There was no statistical heterogeneity (I^2^ = 0%). But as it included various long-acting formulations and was derived from a small number of trials, the finding should be read with caution.

#### 3.6.2. Adverse Events: Long-Acting vs. Sublingual Buprenorphine

**Figure 11:**
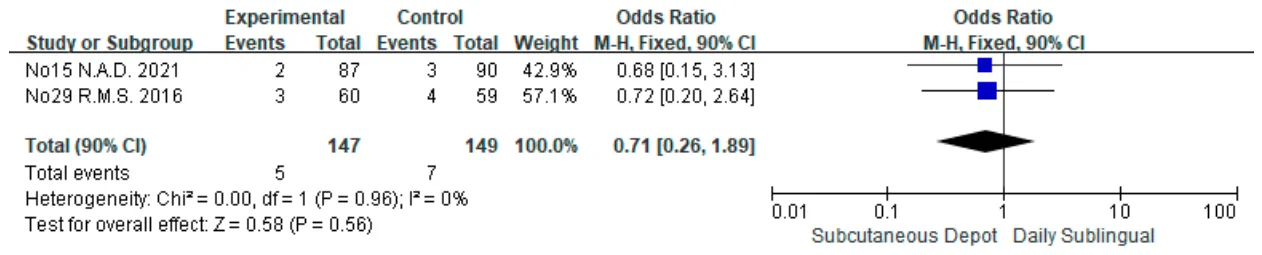
Forest plot of odds ratios comparing long-acting buprenorphine formulations with sublingual buprenorphine for adverse events [8,15]. Squares represent the effect estimates for individual studies, with square size reflecting study weight; the diamond represents the pooled effect estimate, with its width indicating the 90% confidence interval.

As shown in Figure 11, five adverse events were reported in the long-acting formulation group and seven in the sublingual buprenorphine group. This equated to the overall event rate being about 4% of the participants (*n* = 296).

The pooled analysis produced an odds ratio of 0.71 (90% CI [0.26, 1.89]). The confidence interval was large and spanned both an increased and a decreased risk of adverse events with long-acting formulations.

There was no statistical heterogeneity (I^2^ = 0%). But there were very few adverse events reported, and the estimate was very imprecise. There was not enough evidence to determine if there was a difference in adverse event risk between long-acting buprenorphine formulations and sublingual buprenorphine.

### 3.7. Certainty of Evidence

The certainty of evidence for the principal outcomes was assessed using the GRADE approach [11].

For the single 96 mg versus single 32 mg comparison, the certainty of evidence for negative emotional outcomes was rated as low. The evidence was downgraded for the risk of bias and imprecision due to a small number of trials and participants in the analysis. A brief follow-up period has also reduced the scope of application for the results. The certainty of the evidence for the adverse events was also low because few cases had been reported, the confidence interval was large, and concerns about the risk of bias reduced confidence in the comparative safety assessment.

For the single 64 mg versus single 32 mg comparison, the certainty of evidence for negative emotional outcomes was rated as low. The evidence was downgraded for the risk of bias and imprecision due to a small number of trials and participants. Heterogeneity and differences among the studies further reduced the reliability of the pooled estimate. The certainty of the evidence for adverse events was also considered low due to a small number of reported cases and a wide confidence interval.

For the administration-route comparisons, the certainty of evidence for illicit opioid use was rated as low. Due to the small number of trials and the absence of clinically significant uncertainty, the evidence was downgraded for the risk of bias and imprecision. The certainty of the evidence for the adverse event was also low due to a small number of reported cases, a wide confidence interval, and concerns about the risk of bias. 

The key effect estimates and certainty-of-evidence ratings for the principal dose and administration-route comparisons are summarised in Table 2. Detailed Summary-of-Findings tables are provided in Appendix B (Table A1, Table A2 and Table A3).

## 4. Discussion

### 4.1. Principal Findings

This review aimed to collate evidence on dose-related and administration-related emotional and clinical outcomes of buprenorphine treatment for OUD. Overall, pooled estimates of the single-dose comparisons showed a preference for a single 96 mg dose compared with a single 32 mg dose and for a single 64 mg dose compared with a single 32 mg dose for the outcomes of negative emotions measured. However, the results were from a small number of trials and should be viewed with caution.

The point estimate did support the use of long-acting buprenorphine formulations over sublingual buprenorphine for illicit opioid use for the administration route. Due to the few adverse events reported and the wide confidence intervals, no clear differences in adverse-event risk were found for the dose or administration-route comparisons. The overall level of certainty of evidence was low.

### 4.2. Interpretation of Findings

The pooled estimates indicate that single higher doses of buprenorphine may be linked to higher short-term decreases in the negative emotional effects assessed in the trials included. Perhaps the dose-related variation could impact withdrawal-related distress, craving, and other acute symptoms. These mechanisms, however, were not directly addressed in the present meta-analysis and are thus viewed as tentative.

The studies included did not evaluate full psychological stabilisation or long-term recovery but focused on specific emotional outcomes. Thus, the results must not be interpreted to indicate that single larger doses provide better mental health outcomes, adherence to treatment, or relapse prevention. The potential pathways must be explored further through trials.

It is important to note that the dose-comparison results were based on research assessing the acute effects of 32 mg, 64 mg, or 96 mg buprenorphine. These regimens are quite different from the repeated daily maintenance dosing that is commonly used in the maintenance phase of daily OUD treatment. Thus, the findings of these short-term studies should not be interpreted as proof that it is clinically preferable or appropriate to use maintenance treatment at these doses. In addition, a limited number of RCTs were included in these comparisons and results might therefore have limited generalisability.

Finally, the standardised effect estimates should be interpreted with caution. Using standardised mean differences means that outcomes from different scales can be combined, but they do not indicate whether the changes found exceeded a clinically important cut-point on each specific measure. Furthermore, the included trials evaluated somewhat similar, but not identical, emotional constructs, with varying methods of measurement. However, the results of pooling may have been affected by differences in scale properties and sensitivity to short-term change, which may make the results less easily interpretable in clinical practice.

### 4.3. Comparison with Previous Research

The findings are consistent with the previous research indicating that higher doses of buprenorphine are likely to lead to better treatment success, particularly in the case of withdrawal symptoms and cravings [7]. However, the evidence regarding the route of administration is still insufficient. This review showed some benefit for the reduction in illicit drug use with subcutaneous administration. But some research that has compared different administration routes has shown little variation in patient outcomes [3], suggesting that patient preferences and setting may be more important.

Treatment experience may vary by route of administration due to differences in dosing frequency, treatment burden, adherence mandates, and patient preference. Subcutaneous formulations that have a long action will help decrease the need for daily administration and possibly offer a steadier delivery of the drug. However, the trials included did not focus directly on stigma, autonomy, privacy, identity, or social functioning. Thus, the possibility of potential psychosocial effects of the route of administration remains to be explored but was not tested and should be directly studied in future research.

However, depot formulations and implants should not be regarded as interchangeable interventions. They vary in their length of drug administration, how the drug is administered, whether or not they are reversible, the necessity for follow-up, and the degree of burden associated with the procedure. The differences may impact adherence, acceptability, and the patients’ preference, apart from their pharmacological efficacy. Long-acting formulations have potential benefits including lower treatment burden and fewer missed doses, but can be challenging in terms of cost, availability, training for clinicians, procedural requirements, and less flexibility following administration. The ability of a health system to reach these formulations and the capacity of the system may thus affect the ability to replicate the benefits that are seen in the controlled trials in routine clinical practice.

### 4.4. Implications for Clinical Practice and Future Research

Based on the available information, there is not enough evidence to recommend modifying prescribing guidelines or the specific dose or route of administration of buprenorphine. However, the findings point to some key directions for future research designs that explore the emotional and clinical efficacy of treatment for OUD.

Prespecified and consistently defined outcome domains should be used for future trials for better comparability across studies. Ideally, a core outcome set for buprenorphine treatment research should include illicit opioid use, treatment retention, adverse events, outcomes related to relapse, quality of life, and patient-reported emotional outcomes. There should be clearly defined assessment time points and criteria for clinically meaningful change for emotional symptoms, and the instruments used for these assessments should be validated. Sample-size calculations should also be made in advance according to the clinically meaningful difference between the primary outcome and not be based on small exploratory samples. For less common events, such as adverse events and treatment discontinuations, which were very imprecise in the present review, larger samples may be particularly important.

The limited number of trials included in the review is due to the very specific review question and the strict eligibility criteria, and not necessarily to the amount of research on buprenorphine treatment. Past reviews have been quite liberal in incorporating observational studies, non-randomised designs, and/or wider comparisons of buprenorphine and methadone. By contrast, this current review was limited to RCTs that presented extractable comparative data between buprenorphine dose or route of administration. This review cannot rule out publication bias. To minimise this risk, grey literature sources and clinical trial registries were used to supplement the usual bibliographic databases. No other eligible studies were found during the registry searches. Funnel plots and statistical tests for small-study effects were not deemed to be informative, as there were too few studies to contribute to each meta-analysis. Thus, the lack of evidence of publication bias should not be viewed as evidence that publication bias was not present.

### 4.5. Limitations

Several limitations of this review should be acknowledged. First, only five RCTs met the eligibility criteria, and only a small number of studies contributed to each meta-analysis. This reduced the precision and generalisability of the pooled estimates and limited the assessment of heterogeneity and publication bias. Second, the dose-comparison evidence was derived largely from acute single-dose studies, including doses that do not reflect routine maintenance treatment. The findings should therefore not be directly extrapolated to long-term maintenance dosing. Third, the included studies assessed related but not identical emotional outcomes using different instruments and measurement approaches, which limited the direct clinical interpretation of the standardised effect estimates.

Moreover, study screening, data extraction, and risk-of-bias assessment were primarily conducted by a single reviewer rather than independently duplicated by two reviewers. Selected screening decisions and extracted data were verified during the review process, and methodological oversight was provided by M.R.R. and P.M.-M. However, the absence of fully independent duplicate assessment may have increased the possibility of reviewer error or subjective judgement. This limitation should be considered when interpreting the findings.

## 5. Conclusions

Randomised evidence for dose- and administration-related differences in buprenorphine treatment is still limited. Some pooled estimates showed a preference for short-term negative emotional outcomes for single higher-dose buprenorphine and illicit opioid-use outcomes for long-acting buprenorphine formulations. However, these findings were limited to a few trials and were impacted by imprecision and methodological limitations or variations in treatment context.

The one-time doses studied in some of these trials should not be considered standard daily doses, and the results do not justify revisions to current treatment or clinical guidelines. Explicitly powered RCTs in the future should consider using standardised and validated emotional outcome measures along with core clinical outcomes such as illicit opioid use, treatment retention, adverse events, relapses, and longer-term functioning.

## Figures and Tables

**Table 1:** Characteristics and outcome measurement of included studies.

Study	Comparison	Key Outcome(s) Relevant to This Review	Method
Ahmadi and Jahromi [5]	Single-dose buprenorphine: 32 mg vs. 64 mg vs. 96 mg	Anxiety symptoms	Hamilton Anxiety Rating Scale (HAM-A) and clinical interview
Ahmadi et al. [6]	Single-dose buprenorphine: 32 mg vs. 64 mg vs. 96 mg	Suicidal ideation	Beck Scale for Suicidal Ideation (BSSI); DSM-5-based clinical interview
Ahmadi et al. [14]	Single-dose buprenorphine: 32 mg vs. 64 mg vs. 96 mg	Opioid craving	0–10 Visual Analog Scale (VAS) for opioid craving
Lintzeris et al. [8]	Weekly/monthly subcutaneous depot vs. daily sublingual buprenorphine	Mental health symptoms, craving, illicit opioid use, treatment retention, patient-reported outcomes	DASS-21 for depression, anxiety, and stress; craving VAS; urine drug screening (UDS) and TimeLine Follow Back (TLFB) for illicit opioid use; additional PRO instruments including TSQM, SF-36, OSTQoL, SURE, EQ-5D-3L, and ORBIT
Rosenthal et al. [15]	Buprenorphine implant vs. sublingual buprenorphine	Illicit opioid use, craving, withdrawal, treatment retention	Urine drug testing and Timeline Follow Back self-report for illicit opioid use; 100-mm VAS for opioid craving; Subjective Opioid Withdrawal Scale (SOWS) and Clinical Opiate Withdrawal Scale (COWS)

**Table 2:** Summary of key outcomes across studies.

Outcome	Participants (*n*)	Effect Estimate (SMD or OR)	90% CI	Certainty of Evidence (GRADE)
**Mood Regulation** **(96 mg vs. 32 mg)**	51	SMD: −0.66	[−1.00, −0.32]	**Low**
**Mood Regulation** **(64 mg vs. 32 mg)**	296	SMD: −0.34	[−0.68, −0.01]	**Low**
**Illicit Drug Use** **(Long-acting Formulation vs. Sublingual Buprenorphine)**	93	OR: 0.61	[0.39, 0.94]	**Low**
**Adverse Reactions** **(96 mg vs. 32 mg)**	46	OR: 2.7	[0.82, 8.92]	**Low**
**Adverse Reactions** **(64 mg vs. 32 mg)**	93	OR: 0.98	[0.24, 3.92]	**Low**
**Adverse Reactions** **(Long-acting Formulation vs. Sublingual Buprenorphine)**	296	OR: 0.71	[0.26, 1.89]	**Low**

**Note:** Downgraded one level for risk of bias and one level for imprecision.

## Data Availability

All data analysed in this review were obtained from the published studies cited in the article. Extracted data and analytic materials may be made available by the corresponding author upon reasonable request.

## Supplementary Materials

The following supporting information can be downloaded at: https://www.mdpi.com/article/10.3390/healthcare14182995/s1.

## Appendix A

Search Terms and Strategies Used in the Systematic Review

**CENTRAL Search Terms**

1. MeSH descriptor: [Opioid-Related Disorders] explode all trees
2. ((opioid* or opiat*) and (abus* or dependen* or disorder*))
  ,ab,kw (Word variations have been searched)
3. #1 or #2
4. “heroin”
  ,ab,kw (Word variations have been searched)
5. (opioid* or opiat*)
  ,ab,kw (Word variations have been searched)
6. #4 or #5
7. MeSH descriptor: [Buprenorphine] explode all trees
8. MeSH descriptor: [Methadone] explode all trees
9. “buprenorphine”
  ,ab,kw (Word variations have been searched)
10. “methadone”
  ,ab,kw (Word variations have been searched)
11. #7 or #8 or #9 or #10
12. #3 and #6 and #11 from 2013 to 2025, in Trials (Word variations have been searched)

**Embase Search Terms**

1. Opioid-Related Disorders/exp
2. ((opioid* or opiat*) and (abus* or dependen* or disorder*))
  ,ab,kw
3. #1 or #2
4. “heroin”
  ,ab,kw
5. (opioid* or opiat*)
  ,ab,kw
6. #4 or #5
7. Buprenorphine/exp
8. Methadone/exp
9. “buprenorphine”
  ,ab,kw
10. “methadone”
  ,ab,kw
11. #7 or #8 or #9 or #10
12. #3 and #6 and #11
13. “Randomized Controlled Trial”/exp
14. 2013–2025
15. #12 and #13 and #14

**PubMed Search Terms**

1. “Opioid-Related Disorders”[Mesh]
2. ((opioid* OR opiat*) AND (abus* OR dependen* OR disorder*))
3. #1 OR #2
4. “heroin”[tiab]
5. (opioid* OR opiat*)[tiab]
6. #4 OR #5
7. “Buprenorphine”[Mesh]
8. “Methadone”[Mesh]
9. “buprenorphine”[tiab]
10. “methadone”[tiab]
11. #7 OR #8 OR #9 OR #10
12. #3 AND #6 AND #11
13. Randomized Controlled Trial[ptyp]
14. “2013/01/01”[Date—Publication]: “2025/09/30”[Date—Publication]
15. #12 AND #13 AND #14

**APA PsycNET Search Terms**

1. “Opioid-Related Disorders”
2. ((opioid* OR opiat*) AND (abus* OR dependen* OR disorder*))
3. #1 OR #2
4. “heroin”
5. (opioid* OR opiat*)
6. #4 OR #5
7. “Buprenorphine”
8. “Methadone”
9. “buprenorphine”
10. “methadone”
11. #7 OR #8 OR #9 OR #10
12. #3 AND #6 AND #11
13. Randomized Controlled Trial
14. 2013–2025
15. #12 AND #13 AND #14

**Grey Literature Search Terms**

I manually enter the search criteria for grey literature repositories such as CORK, NIDA, and NDARC:

- **Keywords**: opioid-related disorders, buprenorphine, methadone, randomized controlled trial
- **Year setting**: 2013–2025

## Appendix B. Summary-of-Findings Tables

**Table A1:** Effectiveness of buprenorphine (96 mg vs. 32 mg) on negative mood indicators and adverse reactions.

Outcome	Participants (*n*)	Effect Estimate (SMD or OR)	90% CI	Certainty of Evidence (GRADE)
**Negative Mood Indicators** **(96 mg vs. 32 mg)**	51	SMD: −0.66	[−1.00, −0.32]	**Low**
**Adverse Reactions** **(96 mg vs. 32 mg)**	46	OR: 2.7	[0.82, 8.92]	**Low**

**Table A2:** Effectiveness of buprenorphine (64 mg vs. 32 mg) on negative mood indicators and adverse reactions.

Outcome	Participants (*n*)	Effect Estimate (SMD or OR)	90% CI	Certainty of Evidence (GRADE)
**Negative Mood Indicators** **(64 mg vs. 32 mg)**	296	SMD: −0.34	[−0.68, −0.01]	**Low**
**Adverse Reactions** **(64 mg vs. 32 mg)**	93	OR: 0.98	[0.24, 3.92]	**Low**

**Table A3:** Effectiveness of buprenorphine (long-acting formulations vs. sublingual buprenorphine) on illicit drug use and adverse reactions.

Outcome	Participants (*n*)	Effect Estimate (OR)	90% CI	Certainty of Evidence (GRADE)
**Illicit Drug Use** **(Long-acting Formulation vs. Sublingual Buprenorphine)**	93	OR: 0.61	[0.39, 0.94]	**Low**
**Adverse Reactions** **(Long-acting Formulation vs. Sublingual Buprenorphine)**	296	OR: 0.71	[0.26, 1.89]	**Low**
